# Supplementary material for: Acceptance and knowledge of evolutionary theory among third-year university students in Spain
Source: PLoS One. 2020 Sep 3;15(9):e0238345. doi: 10.1371/journal.pone.0238345 (PMC7470367; doi:10.1371/journal.pone.0238345)
Supplement: S1 Table — N is number of students, Nanalysis is the number used in the study (after excluding uncomplete cases). Religiosity represents the percentage of students that claim to be religious practitioner. (DOCX) [file pone.0238345.s003.docx]

**Table S1.** Descriptive parameters of the data questionnaires. N is number of students, N_analysis_ is the number used in the study (after excluding uncomplete cases). Religiosity represents the percentage of students that claim to be religious practitioner.

|  |  |  |  | Complete Questionaries | | | | | | | |
| --- | --- | --- | --- | --- | --- | --- | --- | --- | --- | --- | --- |
| University | Degree | N |  | N_analysis_ | Sex (% Female) | Preuniversity Itinerary (Sci,Tec,Hum,Soc, Art) | Age | Undergraduate (%) | Religiosity (%) | MATE | KEE |
| Universidade de Vigo | Chemistry | 33 |  | 32 | 68.8 | (93.6;3.1;0;0;3.1) | 20.5 ± 1.27 | 93.8 | 22 | 85.5 ± 8.48 | 5.1 ± 1.97 |
|  | History | 8 |  | 8 | 25 | (0;12.5;50.0;37.5;0) | 21.1 ± 2.80 | 100 | 13 | 82.9± 8.48 | 4.7 ± 1.58 |
|  | English Philology | 8 |  | 8 | 87.5 | (12.5;0;62.5;12.5;12.5) | 22.5 ± 2.67 | 87.5 | 13 | 84.6 ± 10.94 | 5.5 ± 2.00 |
|  | Biology | 45 |  | 43 | 67.4 | (100;0;0;0;0) | 20.5 ± 1.53 | 97.7 | 2 | 90.5 ± 6.75 | 6.1 ± 1.60 |
|  |  |  |  |  |  |  |  |  |  |  |  |
| Universidad Autónoma de Madrid | Chemistry | 48 |  | 46 | 54.3 | (93.5;6.5;0;0;0) | 20.6 ± 1.87 | 87 | 20 | 87.5 ± 6.44 | 5.2 ± 1.84 |
|  | History | 11 |  | 10 | 27.3 | (27.3;0;72.7;0;0) | 22.8 ± 8.74 | 100 | 9 | 85.9 ± 10.03 | 4.4 ± 2.01 |
|  | English Philology | 24 |  | 22 | 72.7 | (31.8;0;50;18.2;0) | 21.9 ± 2.47 | 86.4 | 22 | 83.8 ± 7.46 | 4.6 ± 1.62 |
|  | Biology | 43 |  | 43 | 72.1 | (100;0;0;0;0) | 20.1 ± 0.97 | 93 | 21 | 90.3 ± 6.81 | 6.2 ± 1.61 |
|  |  |  |  |  |  |  |  |  |  |  |  |
| Universidad de Alicante | Chemistry | 18 |  | 18 | 27.8 | (100;0;0;0;0) | 20.6 ± 1.20 | 94.4 | 17 | 84.8 ± 8.92 | 5.1 ± 1.60 |
|  | History | 30 |  | 27 | 33.3 | (0;7.4;63;25.9;3.7) | 21.2 ± 2.39 | 92.6 | 15 | 86.7 ± 11.57 | 4.6 ± 1.97 |
|  | English Philology | 11 |  | 10 | 50 | (10;0;50;40;0) | 20.6 ± 1.07 | 100 | 10 | 84.1 ± 12.04 | 4.2 ± 1.14 |
|  | Biology | 44 |  | 44 | 40.9 | (100;0;0;0;0) | 20.6 ± 1.80 | 97.7 | 2 | 91.8 ± 7.29 | 6.4 ± 1.62 |
|  |  |  |  |  |  |  |  |  |  |  |  |
| Universidad de Sevilla | Chemistry | 24 |  | 24 | 48.8 | (96;4;0;0;0) | 21.8 ± 2.52 | 92 | 24 | 91.5 ± 7.63 | 5.6 ± 1.53 |
|  | History | 17 |  | 17 | 17.6 | (11.8;11.8;41.2;35.3;0) | 22.5 ± 5.81 | 94.1 | 35 | 89.2 ± 7.40 | 4.5 ± 2.18 |
|  | English Philology | 7 |  | 7 | 100 | (0;0;100;0;0) | 21.0 ± 0.58 | 57.1 | 33 | 87.3 ± 7.87 | 4.0 ± 0.82 |
|  | Biology | 33 |  | 33 | 69.7 | (100;0;0;0;0) | 20.4 ± 0.89 | 97 | 12 | 89.6 ± 5.95 | 6.0 ± 1.74 |
|  |  |  |  |  |  |  |  |  |  |  |  |
| Universidad de Granada | Chemistry | 23 |  | 23 | 69.6 | (82.6;17.4;0;0;0) | 20.9 ± 1.41 | 91.3 | 30 | 82.8 ± 6.77 | 4.7 ± 1.37 |
|  | History | 32 |  | 30 | 43.3 | (3.3;6.7;46.7;40;3.3) | 23.6 ± 4.13 | 66.7 | 13 | 82.9 ± 7.42 | 4.4 ± 1.59 |
|  | English Philology | 18 |  | 17 | 70.6 | (5.9;0;88.2;5.9;0) | 21.1 ± 1.25 | 70.6 | 24 | 81.2 ± 9.65 | 4.1 ± 1.75 |
|  | Biology | 59 |  | 59 | 64.4 | (100;0;0;0;0) | 20.6 ± 1.33 | 71.2 | 5 | 91.0 ± 6.70 | 6.4 ± 1.60 |
|  |  |  |  |  |  |  |  |  |  |  |  |
| Universidad de Valencia | Chemistry | 18 |  | 17 | 52.9 | (94.1;5.9;0;0;0) | 22.7 ± 5.18 | 100 | 6 | 87.4 ± 8.29 | 5.3 ± 1.86 |
|  | History | 13 |  | 13 | 15.4 | (15.4;0;53.8;30.8;0) | 23.6 ± 11.27 | 100 | 8 | 87.2 ± 5.44 | 5.4 ± 1.80 |
|  | English Philology | 33 |  | 30 | 73.3 | (13.3;0;76.7;3.3;6.7) | 22.3 ± 6.05 | 83.3 | 20 | 82.9 ± 7.41 | 4.6 ± 1.77 |
|  | Biology | 31 |  | 31 | 54.8 | (96.8;0;3.2;0;0) | 21.0 ± 5.03 | 90.3 | 10 | 89.5 ± 6.50 | 7.1 ± 2.04 |
|  |  |  |  |  |  |  |  |  |  |  |  |
| Universidad Complutense de Madrid | Chemistry | 26 |  | 26 | 61.5 | (88.5;11.5;0;0;0) | 20.7 ± 1.46 | 96.2 | 8 | 85.9 ± 9.64 | 5.5 ± 1.77 |
|  | History | 9 |  | 9 | 10 | (10;0;70;20;0) | 28.0 ± 12.30 | 55.5 | 30 | 86.9 ± 8.18 | 6.1 ±1.59 |
|  | English Philology | 21 |  | 21 | 71.4 | (4.8;9.5;71.4;14.3;0) | 20.2 ± 1.36 | 90.5 | 14 | 83.9 ± 11.02 | 4.8 ± 2.11 |
|  | Biology | 29 |  | 25 | 84 | (100;0;0;0;0) | 20.0 ± 0.35 | 92 | 24 | 85.2 ± 11.54 | 6.9 ± 1.94 |
|  |  |  |  |  |  |  |  |  |  |  |  |
| Universidad de Salamanca | Chemistry | 42 |  | 42 | 64.3 | (95.2;4.8;0;0;0) | 22.2 ± 5.93 | 78.6 | 31 | 82.4 ± 8.55 | 4.6 ± 1.55 |
|  | History | 4 |  | 3 | 33.3 | (66.7;0;33.3;0;0) | 22.3 ± 2.08 | 33.3 | 0 | 87.7 ± 11.85 | 7.0 ± 1.73 |
|  | English Philology | 30 |  | 30 | 86.7 | (6.7;3.3;76.7;13.3;0) | 20.2 ± 0.85 | 96.7 | 27 | 83.0 ± 9.76 | 4.0 ± 1.53 |
|  | Biology | 14 |  | 14 | 78.6 | (100;0;0;0;0) | 20.3 ± 1.38 | 78.6 | 7 | 88.4 ± 8.57 | 6.1 ± 1.96 |
|  |  |  |  |  |  |  |  |  |  |  |  |
| Universidade de Santiago de Compostela | Chemistry | 35 |  | 32 | 43.8 | (96.9;3.1;0;0;0) | 21.1 ± 1.33 | 87.5 | 13 | 87.5 ± 11.53 | 5.4 ± 1.93 |
|  | History | 21 |  | 20 | 40 | (5;0;65;30;0) | 20.1 ± 0.79 | 95 | 10 | 91.0 ± 5.63 | 5.1 ± 1.50 |
|  | English Philology | 45 |  | 42 | 83.3 | (11.9;2.4;71.4;11.9;2.4) | 20.6 ± 1.12 | 85.7 | 7 | 86.5 ± 8.26 | 4.5 ± 1.43 |
|  | Biology | 34 |  | 34 | 55.9 | (100;0;0;0;0) | 20.1 ± 0.70 | 94.1 | 6 | 90.1 ± 6.60 | 7.4 ± 1.35 |
|  |  |  |  |  |  |  |  |  |  |  |  |
| Universitat de les Illes Balears | Chemistry | 16 |  | 13 | 61.5 | (100;0;0;0;0) | 21.1 ± 1.28 | 100 | 0 | 91.2 ± 5.51 | 5.5 ± 1.33 |
|  | History | 11 |  | 11 | 54.5 | (0;0;63.6;36.4;0) | 24.2 ± 8.51 | 90.9 | 0 | 86.7 ±8.45 | 4.2 ± 1.40 |
|  | English Philology | 17 |  | 16 | 93.8 | (6.3;6.3;75;12.5;0) | 24.8 ± 10.53 | 87.5 | 0 | 82.4 ± 9.19 | 4.2 ± 1.80 |
|  | Biology | 29 |  | 28 | 60.7 | (100;0;0;0;0) | 20.3 ± 1.12 | 85.7 | 0 | 91.2 ± 7.24 | 6.1 ± 1.59 |
|  |  |  |  |  |  |  |  |  |  |  |  |
| Erasmus | Chemistry | 1 |  | 1 | 0 | (100;0;0;0;0) | 22 | 100 | 0 | 82 | 5 |
|  | History | 10 |  | 10 | 50 | (20;0;50;30;0) | 22.4 ± 6.22 | 80 | 40 | 84.7 ±13.03 | 4.2 ± 1.87 |
|  | English Philology | 12 |  | 9 | 77.8 | (11.1;0;88.9;0;0) | 20.7 ± 1.22 | 100 | 22 | 81.2 ±5.17 | 4.4 ± 1.67 |
|  | Biology | 2 |  | 2 | 50 | (100;0;0;0;0) | 21.0 ± 1.41 | 100 | 0 | 90.0 ± 5.66 | 5.5 ± 0.71 |
