# Supplementary material for: Acceptance and knowledge of evolutionary theory among third-year university students in Spain
Source: PLoS One. 2020 Sep 3;15(9):e0238345. doi: 10.1371/journal.pone.0238345 (PMC7470367; doi:10.1371/journal.pone.0238345)
Supplement: S2 Table — Course is the year of the given lecture. Total content is the number of themes of the lecture, while subject content is the number of those related with Evolution. Probability is the ratio between the former two, while Evolution credits is the sum of the credits x probability. (DOCX) [file pone.0238345.s004.docx]

**Table S2.** Description of the curricula of the Biology degrees per University. Course is the year of the given lecture. Total content is the number of themes of the lecture, while subject content is the number of those related with Evolution. Probability is the ratio between the former two, while Evolution credits is the sum of the credits x probability:

| University | Lecture | Course | Semester | Subject content | Total content | Probability of Evolution content | Credits ECTS | Evolution Credits |
| --- | --- | --- | --- | --- | --- | --- | --- | --- |
| Vigo | Evolution | 1 | 1 | 19 | 19 | 1 | 6 | 13.97 |
|  | Botany I | 1 | 1 | 1 | 20 | 0.05 | 6 |  |
|  | Microbiology I | 1 | 1 | 1 | 39 | 0.02564103 | 6 |  |
|  | Zoology I | 2 | 1 | 1 | 19 | 0.05263158 | 6 |  |
|  | Microbiology II | 3 | 2 | 2 | 10 | 0.2 | 6 |  |
|  | Genetics II | 3 | 1 | 6 | 6 | 1 | 6 |  |
|  |  |  |  |  |  |  |  |  |
| Autónoma de Madrid | Zoology I | 1 | Annual | 2 | 33 | 0.06060606 | 12 | 13.3 |
|  | Geology | 1 | Annual | 3 | 14 | 0.21428571 | 9 |  |
|  | Botany | 2 | Annual | 1 | 37 | 0.02702703 | 12 |  |
|  | Microbiology | 2 | Annual | 2 | 44 | 0.04545455 | 12 |  |
|  | History of Biology and Evolution | 2 | 1 | 6 | 7 | 0.85714286 | 6 |  |
|  | Genetics | 2 | Annual | 5 | 15 | 0.33333333 | 12 |  |
|  | Ecology | 3 | Annual | 1 | 19 | 0.05263158 | 12 |  |
|  |  |  |  |  |  |  |  |  |
| Alicante | Biology | 1 | 1 | 5 | 20 | 0.25 | 6 | 8.19 |
|  | Genetics | 1 | 2 | 14 | 44 | 0.31818182 | 6 |  |
|  | Geology | 1 | 2 | 3 | 10 | 0.3 | 6 |  |
|  | Zoology | 2 | 1 | 3 | 30 | 0.1 | 6 |  |
|  | Botany | 2 | 1 | 1 | 20 | 0.05 | 6 |  |
|  | Microbiology | 2 | 1 | 1 | 9 | 0.11111111 | 6 |  |
|  | Plant Biodiversity | 2 | 2 | 1 | 23 | 0.04347826 | 6 |  |
|  | Population Ecology | 3 | 2 | 1 | 15 | 0.06666667 | 6 |  |
|  | Advanced Microbiology | 3 | 2 | 1 | 8 | 0.125 | 6 |  |
|  |  |  |  |  |  |  |  |  |
| Sevilla | Genetics I | 2 | 1 | 8 | 15 | 0.53333333 | 6 | 5.08 |
|  | Botany II | 2 | 2 | 1 | 8 | 0.125 | 6 |  |
|  | Genetics II | 2 | 2 | 1 | 13 | 0.07692308 | 6 |  |
|  | Microbiology | 3 | 1 | 1 | 9 | 0.11111111 | 6 |  |
|  |  |  |  |  |  |  |  |  |
| Granada | Conceptual Development of Biology | 1 | 1 | 2 | 10 | 0.2 | 6 | 14.53 |
|  | Genetics I | 1 | 2 | 8 | 8 | 1 | 6 |  |
|  | Evolutionary Biology | 1 | 2 | 18 | 18 | 1 | 6 |  |
|  | Zoology | 2 | Annual | 3 | 27 | 0.11111111 | 12 |  |
|  |  |  |  |  |  |  |  |  |
| Valencia | Biology | 1 | 1 | 2 | 26 | 0.07692308 | 6 | 14.16 |
|  | The Tree of Life | 1 | 1 | 8 | 8 | 1 | 6 |  |
|  | Genetics | 2 | Annual | 3 | 25 | 0.12 | 10 |  |
|  | Main Evolutionary Transitions | 2 | 2 | 4 | 4 | 1 | 6 |  |
|  | Paleontology | 2 | 1 | 1 | 12 | 0.08333333 | 6 |  |
|  |  |  |  |  |  |  |  |  |
| Complutense | Genetics | 2 | Annual | 2 | 15 | 0.13333333 | 12 | 17.36 |
|  | Physical Anthropology | 3 | 1 | 10 | 21 | 0.47619048 | 6 |  |
|  | Evolutionary Biology | 3 | Annual | 21 | 21 | 1 | 12 |  |
|  | Ethology | 3 | 1 | 3 | 20 | 0.15 | 6 |  |
|  |  |  |  |  |  |  |  |  |
| Salamanca | Evolutionary Biology | 1 | 2 | 8 | 8 | 1 | 6 | 8.78 |
|  | Plant Histology and Cell Biology | 1 | 2 | 1 | 21 | 0.04761905 | 6 |  |
|  | Geology | 1 | 1 | 2 | 19 | 0.10526316 | 6 |  |
|  | Genetics | 2 | Annual | 9 | 29 | 0.31034483 | 6 |  |
|  |  |  |  |  |  |  |  |  |
| Santiago de Compostela | Foundations of Biology | 1 | 1 | 5 | 18 | 0.27777778 | 6 | 14.75 |
|  | Genetics | 2 | 1 | 4 | 14 | 0.28571429 | 6 |  |
|  | Evolutionary Genetics | 3 | 1 | 18 | 18 | 1 | 6 |  |
|  | Biological Anthropology | 3 | 1 | 17 | 19 | 0.89473684 | 6 |  |
|  |  |  |  |  |  |  |  |  |
| Islas Baleares | Geology | 1 | 2 | 1 | 9 | 0.11111111 | 6 | 11.91 |
|  | Microbiology | 2 | 1 | 1 | 8 | 0.125 | 6 |  |
|  | Genetics | 2 | 1 | 4 | 19 | 0.21052632 | 6 |  |
|  | Zoology I | 2 | 2 | 1 | 12 | 0.08333333 | 6 |  |
|  | Evolution | 2 | 2 | 12 | 12 | 1 | 6 |  |
|  | Zoology II | 3 | 1 | 5 | 11 | 0.45454545 | 6 |  |
