# Supplementary material for: Acceptance and knowledge of evolutionary theory among third-year university students in Spain
Source: PLoS One. 2020 Sep 3;15(9):e0238345. doi: 10.1371/journal.pone.0238345 (PMC7470367; doi:10.1371/journal.pone.0238345)
Supplement: S3 Table — Pink shaded for those significant (without multitest correction) and Red shaded for those significant after SGoF multitest adjustment. (DOCX) [file pone.0238345.s005.docx]

**Table S3.** Pairwise chi-square tests for the different variables pairs per university. Pink shaded for those significant (without multitest correction) and Red shaded for those significant after SGoF multitest adjustment.

| University |  | Sex | Religious | Level | preuniversity | Faculty |
| --- | --- | --- | --- | --- | --- | --- |
|  | Sex |  | 2.5 | 3.4 | 3.9 | 11.1 |
|  | Religious |  |  | 3 | 3.3 | 7.7 |
| Vigo | Level |  |  |  | 4.9 | 2.3 |
|  | preuniversity |  |  |  |  | 77.3 |
|  |  |  |  |  |  |  |
|  | Sex |  | 5.9 | 3.1 | 5 | 12.4 |
|  | Religious |  |  | 1.8 | 2.1 | 1.3 |
| Autónoma | Level |  |  |  | 2.3 | 3.2 |
|  | Itinerary |  |  |  |  | 86.3 |
|  |  |  |  |  |  |  |
|  | Sex |  | 0.4 | 4 | 4.8 | 3.6 |
|  | Religious |  |  | 0.1 | 2.6 | 5.4 |
| Alicante | Level |  |  |  | 0.9 | 2.0 |
|  | Itinerary |  |  |  |  | 125.8 |
|  |  |  |  |  |  |  |
|  | Sex |  | 0.3 | 3.6 | 12.7 | 24.2 |
|  | Religious |  |  | 0.1 | 7.4 | 4.2 |
| Sevilla | Level |  |  |  | 4 | 7.8 |
|  | Itinerary |  |  |  |  | 89.1 |
|  |  |  |  |  |  |  |
|  | Sex |  | 5 | 2.5 | 5.1 | 13.1 |
|  | Religious |  |  | 0.1 | 5.7 | 2.7 |
| Valencia | Level |  |  |  | 3.7 | 7.4 |
|  | Itinerary |  |  |  |  | 90.3 |
|  |  |  |  |  |  |  |
|  | Sex |  | 0.4 | 6.2 | 8.4 | 21.6 |
|  | Religious |  |  | 0.1 | 0.3 | 3.9 |
| Complutense | Level |  |  |  | 10.6 | 11.3 |
|  | Itinerary |  |  |  |  | 91.4 |
|  |  |  |  |  |  |  |
|  | Sex |  | 0.1 | 7.4 | 9.7 | 8.1 |
|  | Religious |  |  | 1.6 | 2.9 | 5.6 |
| Salamanca | Level |  |  |  | 5.5 | 11 |
|  | Itinerary |  |  |  |  | 91.7 |
|  |  |  |  |  |  |  |
|  | Sex |  | 1.1 | 0.8 | 5.2 | 5.5 |
|  | Religious |  |  | 1.1 | 7 | 10.2 |
| Granada | Level |  |  |  | 2.4 | 5.6 |
|  | Itinerary |  |  |  |  | 165.1 |
|  |  |  |  |  |  |  |
|  | Sex |  | - | 0.4 | 2.5 | 7.1 |
|  | Religious |  |  | - | - | - |
| Baleares | Level |  |  |  | 3.2 | 3.4 |
|  | Itinerary |  |  |  |  | 139.6 |
|  |  |  |  |  |  |  |
|  | Sex |  | 1.3 | 0.4 | 9.2 | 21.8 |
|  | Religious |  |  | 2.5 | 1.7 | 1.2 |
| Santiago | Level |  |  |  | 1.5 | 2.4 |
|  | Itinerary |  |  |  |  | 91.7 |
|  |  |  |  |  |  |  |
|  |  |  |  |  |  |  |
|  |  |  |  |  |  |  |
|  |  |  |  | SGoF corrected | |  |
|  | Sex |  | 0 | 0 | 0 | 4 |
|  | Religious |  |  | 0 | 0 | 0 |
| summary | Level |  |  |  | 0 | 1 |
|  | Itinerary |  |  |  |  | 10 |
|  |  |  |  |  |  |  |
|  |  |  |  |  |  |  |
|  |  |  | * |  |  |  |
|  |  |  | ** |  |  |  |
|  |  |  | *** |  |  |  |
