# Supplementary material for: Acceptance and knowledge of evolutionary theory among third-year university students in Spain
Source: PLoS One. 2020 Sep 3;15(9):e0238345. doi: 10.1371/journal.pone.0238345 (PMC7470367; doi:10.1371/journal.pone.0238345)
Supplement: S4 Table — (DOCX) [file pone.0238345.s006.docx]

**Table S4.** Analysis of the homogeneity chi-square test for demographic variables within University.

| Factor | G test | DF | Prob. | Trend | Deviation |
| --- | --- | --- | --- | --- | --- |
| Sex | 44.7 | 18 | < 0.001 | 60% women | Alicante (40%) |
|  |  |  |  |  |  |
| Academic Level | 42.8 | 18 | 0.001 | 88% bachelor | Granada (74%) |
|  |  |  |  |  |  |
| Itinerary | 61.9 | 36 | 0.005 | 66% science | Vigo (80% science) |
|  |  |  |  | 3% technology |  |
|  |  |  |  | 24% humanity |  |
|  |  |  |  | 7% social |  |
|  |  |  |  | 1% art |  |
|  |  |  |  |  |  |
| Faculty | 136.8 | 27 | < 0.001 | 28% Chemestry | Several |
|  |  |  |  | 15% History |  |
|  |  |  |  | 21% Philology |  |
|  |  |  |  | 36% Biology |  |
|  |  |  |  |  |  |
| Religiosity | 40.6 | 9 | < 0.001 | 14% religious | Baleares (0%) |
|  |  |  |  |  | Salamanca (25%) |
|  |  |  |  |  | Sevilla (22%) |
