# Supplementary material for: Acceptance and knowledge of evolutionary theory among third-year university students in Spain
Source: PLoS One. 2020 Sep 3;15(9):e0238345. doi: 10.1371/journal.pone.0238345 (PMC7470367; doi:10.1371/journal.pone.0238345)
Supplement: S1 Fig — (PDF) [file pone.0238345.s007.pdf]

EVOLUTION SURVEY

Coordinator (Emilio Rolán-Alvarez; Universidad de Vigo; rolan@uvigo.es)

* Required

1. **Verification code** *

2. **Sex** *
   Mark only one oval.
   - Man
   - Woman
   - I prefer not to choose

3. **Age** *

4. **Academic level attained** *
   Mark only one oval.
   - Secondary-school
   - Graduate
   - Doctor

5. **Secondary school itinerary** *
   Mark only one oval.
   - Sciences
   - Technology
   - Humanities
   - Social sciences
   - Art

6. **University** *
Mark only one oval.

- Valencia
- Alicante
- Juan Carlos
- Alcalá
- Santiago
- Islas Baleares
- Jaen
- Córdoba
- Extremadura
- León
- de Barcelona
- País Vasco
- La Laguna
- erasmus o similares

7. **Grade** *
Mark only one oval.

- Biology
- Chemistry
- Languages
- History

8. **¿Are you a religious practitioner?**
Mark only one oval.

- Yes
- No
- Other: ____________________

Skip to question 9.

Secction I

Answer each of the statements by clicking on the answer that seems appropriate to you.

9. **1. Organisms existing today are the result of evolutionary processes that have occurred over millions of years.**
Mark only one oval.

- Strongly disagree
- Disagree
- Undecided
- Agree
- Strongly agree

10. **2. The theory of evolution is incapable of being scientifically tested.**
Mark only one oval.

- Strongly disagree
- Disagree
- Undecided
- Agree
- Strongly agree

11. **3. Modern humans are the product of evolutionary processes that have occurred over millions of years.**
Mark only one oval.

- Strongly disagree
- Disagree
- Undecided
- Agree
- Strongly agree

12. **4. The theory of evolution is based on speculation and not valid scientific observation and testing.**
Mark only one oval.

- Strongly disagree
- Disagree
- Undecided
- Agree
- Strongly agree

13. **5. Most scientists accept evolutionary theory to be a scientifically valid theory.**
Mark only one oval.

- Strongly disagree
- Disagree
- Undecided
- Agree
- Strongly agree

14. **6. The available data are ambiguous (unclear) as to whether evolution actually occurs.**
Mark only one oval.

- Strongly disagree
- Disagree
- Undecided
- Agree
- Strongly agree

15. **7. The age of the hearth is less than 20,000 years**
Mark only one oval.

- Strongly disagree
- Disagree
- Undecided
- Agree
- Strongly agree

16. **8. There is a significant body of data that supports evolutionary theory.**
Mark only one oval.

- Strongly disagree
- Disagree
- Undecided
- Agree
- Strongly agree

17. **9. Organisms exist today essentially in the same form in which they always have.**
Mark only one oval.

- Strongly disagree
- Disagree
- Undecided
- Agree
- Strongly agree

18. **10. Evolution is not a scientifically valid theory.**
Mark only one oval.

- Strongly disagree
- Disagree
- Undecided
- Agree
- Strongly agree

19. **11. The age of the earth is at least 4 billions years.**
Mark only one oval.

- Strongly disagree
- Disagree
- Undecided
- Agree
- Strongly agree

20. **12. Current evolutionary theory is the result of sound scientific research and methodology.**
Mark only one oval.

- Strongly disagree
- Disagree
- Undecided
- Agree
- Strongly agree

21. **13. Evolutionary theory generates testable predictions with respect to the characteristics of life.**
Mark only one oval.

- Strongly disagree
- Disagree
- Undecided
- Agree
- Strongly agree

22. **14. The theory of evolution cannot be corrected since it disagrees with the Biblical account of creation.**
Mark only one oval.

- Strongly disagree
- Disagree
- Undecided
- Agree
- Strongly agree

23. **15. Humans exist today in essentially the same form in which they always have.**
Mark only one oval.

- Strongly disagree
- Disagree
- Undecided
- Agree
- Strongly agree

24. **16. Evolutionary theory is supported by factual historical and laboratory data.**
Mark only one oval.

- Strongly disagree
- Disagree
- Undecided
- Agree
- Strongly agree

25. **17. Much of the scientific community doubts if evolution occurs.**
Mark only one oval.

- Strongly disagree
- Disagree
- Undecided
- Agree
- Strongly agree

26. **18. The theory of evolution brings meaning to the diverse characteristics and behavior observed in living forms.**
Mark only one oval.

- Strongly disagree
- Disagree
- undecided
- Agree
- Strongly agree

27. **19. With few exceptions, organisms on earth came into existence at about the same time.**
Mark only one oval.

- Strongly disagree
- Disagree
- Undecided
- Agree
- Strongly agree

28. **20. Evolution is a scientifically valid theory.**
Mark only one oval.

- Strongly disagree
- Disagree
- Undecided
- Agree
- Strongly agree

Section II

Click on the right answer

29. **21. Which of the following observations helps to understand the theory of evolution?**
Mark only one oval.

- Artificial selection (selective enhancement) as an analogue of natural selection.
- Comparative genomics, by comparing and quantifying the differences and similarities of DNA between species
- Vestigial structures that serve no apparent purpose.
- Comparative embryology, where the evolutionary history of similar structures can often be followed.
- All of the above evidences supports the theory of evolution.

30. **22. Recently, resistance to a wide variety of insecticides has appeared in various species of insects. Why?**
Mark only one oval.

- Mutations are increasing
- Humans alter the environment of these organisms and they evolve by natural selection.
- New species are not evolving, only strains or varieties, it is not evolution by natural selection.
- Humans improve their healthcare strategies and organisms try to keep up to date
- Insects are smarter than humans.

31. **23. Which of the following cases would have a greater effectiveness in evolutionary terms?**
Mark only one oval.

- A very efficient lion capturing preys but without offspring.
- A lion that has many offspring and 8 of them have become adults.
- A lion that survived an illness and had 3 offspring.
- A lion who had a harem of female lions and had only one offspring.
- A lion that takes good care of its offspring and 3 of them have become adults.

32. **24. How would a biologist explain why a species of bird has evolved a larger beak?**
Mark only one oval.

- Larger beak size appears by mutation in each member of the bird population.
- The ancestors of this species found a new food source (larger seeds) and needed to develop better beaks to adapt to the new food.
- Some members of the ancestral bird population had larger beaks than others, as the larger beaks were advantageous in the ancestral environment, they were able to leave more descendants so that the large beak character changed its frequency.
- An ancestor of the species found bigger seeds and, trying to split them, the beak developed more. It transmitted this bigger beak to its descendants and with time to the whole population.
- None of the previous ones.

33. **25. Which of the following statements about natural selection is true?**
Mark only one oval.

- Natural selection causes variation to arise within a population.
- All individuals in a population have the same probability of survival and reproduction. Survival depends on luck
- Natural selection occurs in those individuals in a population who are best adapted, surviving better and leaving more descendants.
- Natural selection leads to extinction.
- Natural selection allows the probability of survival of certain individuals to increase depending of the variation. Variation comes from outside the population.

34. **26. All organisms share the same genetic code; this circumstance is evidence that...**
Mark only one oval.

- Evolution is happening now.
- Convergent evolution has occurred.
- Evolution occurs gradually.
- All organisms are descended from a single ancestor.
- Life began millions of years ago

35. **27. Which of the following statements regarding evolution by natural selection is FALSE?**
Mark only one oval.

- Natural selection acts on individuals.
- Natural selection is a random process.
- Very small selective effects can produce very large population changes during enough time.
- Natural selection can cause the elimination of certain alleles in a population.
- Mutation is the ultimate source of variation on which natural selection acts.

36. **28. A change in the genetic material of an organism over time is...**
Mark only one oval.

- Adaptive radiation
- Biological evolution
- Lamarkist evolution
- Natural selection
- Genetic recombinatiion

37. **29. Which of the followings is the (ultimate) source of the new variation in natural populations?**

Mark only one oval.

- Recombination
- Hybridization
- Gene flow
- Mutation
- Natural selection

38. **30. Which of the following statements about the relationship between evolution and natural selection is correct?**

Mark only one oval.

- Natural selection is a mechanism that can produce evolution.
- Natural selection is a process that can produce small-scale changes in populations, while evolution produces large-scale changes.
- Natural selection is a random process, while evolution produces changes in a given direction.
- Natural selection is the differential survival of populations or groups, resulting in the evolution of individual organisms.
- They are equivalent terms describing the same process.

Powered by
Google Forms
